# Degradation Rates and Bacterial Community Compositions Vary among Commonly Used Bioplastic Materials in a Brackish Marine Environment

**DOI:** 10.1021/acs.est.2c06280

**Published:** 2022-10-21

**Authors:** Eeva L. Eronen-Rasimus, Pinja P. Näkki, Hermanni P. Kaartokallio

**Affiliations:** † Department of Microbiology, University of Helsinki, Viikinkaari 9, 00790 Helsinki, Finland; ‡ Marine Research Centre, Finnish Environment Institute, Agnes Sjöbergin katu 2, 00790 Helsinki, Finland

**Keywords:** bioplastic, marine litter, degradation, biodegradation, Baltic Sea, bacterial communities, 16S rRNA gene

## Abstract

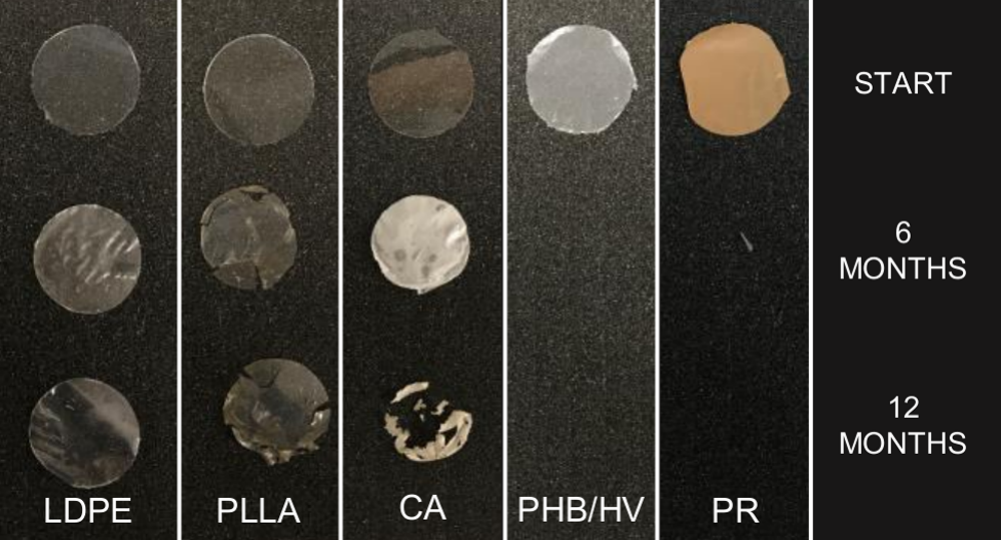

Plastic pollution threatens both terrestrial and aquatic ecosystems. As a result of the pressures of replacing oil-based materials and reducing the accumulation of litter in the environment, the use of bioplastics is increasing, despite little being known about their accurate biodegradation in natural conditions. Here, we investigated the weight attrition and degradation behavior of four different bioplastic materials compared to conventional oil-based polyethylene during a 1-year *in situ* incubation in the brackish Baltic Sea and in controlled 1 month biodegradation experiments in the laboratory. Bacterial communities were also investigated to verify whether putative plastic-degrading bacteria are enriched on bioplastics. Poly-l-lactic acid showed no signs of degradation, whereas poly(3-hydroxybutyrate/3-hydroxyvalerate) (PHB/HV), plasticized starch (PR), and cellulose acetate (CA) degraded completely or almost completely during 1-year *in situ* incubations. In accordance, bacterial taxa potentially capable of using complex carbon substrates and belonging, e.g., to class Gammaproteobacteria were significantly enriched on PHB/HV, PR, and CA. An increase in gammaproteobacterial abundance was also observed in the biodegradation experiments. The results show substantial differences in the persistence and biodegradation rates among bioplastics, thus highlighting the need for carefully selecting materials for applications with risk of becoming marine litter.

## Abstract 2

Limited knowledge is available on the accurate biodegradation of biodegradable plastics. In this study, we show with 1 year *in situ* incubations and 1 month biodegradation experiments that not all bioplastics are biodegradable in brackish marine environments.

## Introduction

Plastic pollution is a ubiquitous threat to nature, on both land and sea. Millions of tons of mismanaged plastic waste are estimated to enter the ocean every year,^1^ and because most plastic materials are very durable,^2^ their continuous loading may potentially result in accumulation with deleterious and far-reaching consequences. Because the global use of plastics is constantly increasing,^3^ there is a pressing need to prevent their transport to the sea and mitigate their effects on these ecosystems.

Bioplastics, i.e., plastics that are biodegradable, bio-based, or both,^4^ are promoted as more sustainable alternatives to alleviate the long-lasting and harmful impacts of plastics on the environment and to reduce the dependence of the plastic industry on crude oil as a primary raw material. They are often made from bio-based, renewable carbon sources, such as cellulose, starch, and polyhydroxyalkanoates (PHAs).^5,6^ Although bioplastics currently make up only about 1% of total plastic production, they are expected to become more popular in many applications,^4^ which increases their probability of ending up in the marine environment. For example, a recent European Union legislation targets single-use plastic as the form most commonly becoming marine litter. For product categories among common marine litter items, such as straws and single-use cutlery, only the use of non-modified biopolymers (e.g., cellulose) is currently allowed until further research on environmental safety of biodegradable alternatives is available^7^ (EC/P 2021).

When in the sea, the degradation of plastics is divided into abiotic and biotic degradation (reviewed in ref (8)). In short, usually long polymer chains are first abiotically broken into shorter molecules [e.g., by ultraviolet (UV) radiation, wave action, and salts] and then subjected to further biodegradation (assimilation and mineralization) by microbes (reviewed in refs (8−10)). The biodegradation of plastics is dependent upon the material properties as well as specific abiotic and biotic conditions that, for most plastic materials, are rarely met in the open environment.^11^ Fully biodegradable materials are mineralized, depending upon whether oxygen is available, into carbon dioxide (CO_2_), mineral salts, and microbial biomass (aerobic) or CO_2_, methane, mineral salts, and microbial biomass (anaerobic) when exposed to natural conditions.^11^

It has been estimated that marine microbes play a negligible role in degradation of petrochemical plastics.^9^ However, they are known to leach dissolved organic carbon (DOC), therefore promoting bacterial activity in the oceans.^12−15^ The debate on whether typical microbial communities forming on plastic surfaces in seawater referred to as plastisphere^16^) communities vary between polymer types and/or among other surfaces is still ongoing. The consensus is forming toward plastisphere community differences being more dependent upon environmental conditions than polymer type.^9,17−20^ In contrast, upon biodegradable plastic materials, both bacterial community composition and/or bacterial activity differentiate compared to reference materials, indicating that certain bioplastics could actually harbor communities capable of biodegradation. However, the amount of literature on biodegradable plastics is still limited and have been concentrated mostly on polylactic acid (PLA) and polyhydroxybutyrate (PHB).^21−24^ Differences in biodegradability between polymer types have also been reported; e.g., in seawater, PHB is more prone to biodegradation than PLA.^22,24−27^ These studies have provided valuable information on bioplastic behavior and possible degradation in marine environments; however, studies with multiple bioplastic materials combining methodologies on actual biodegradation (biological oxygen demand) and *in situ* experiments, including both weight attrition and bacterial community composition, are lacking. Also, bacterial community dynamics on bioplastic materials on a yearly scale are still needed. Because bioplastics are viewed as a replacement for recalcitrant fossil-based materials, assessment of their natural biodegradability is needed to avoid unintentional transition from non-degradable conventional plastics to non-degradable bio-based plastics. Furthermore, it must be ensured that they do not otherwise harm the environment, for example, by being toxic to organisms or affecting the important biogeochemical processes in the sea^28,29^

Here, we investigated the biodegradation of various bioplastic (in this case, bio-based and biodegradable) materials in the brackish northern Baltic Sea (salinity at the incubation site of 4–7), both *in situ* and *ex situ*, by focusing on their weight attrition during long-term incubations in the sea and their complete biodegradation in controlled 1-month laboratory experiments. In addition, the bacterial community composition was investigated to determine the presence of putative plastic-degrading bacterial taxa.

## Materials and Methods

### Preparation of the Sampling Racks

We studied one conventional plastic film [ET311150/1 low-density polyethylene (LDPE)], with a thickness of 0.05 mm, and four bio-based, biodegradable plastic films: (1) AC311051 cellulose acetate (CA), with a thickness of 0.05 mm, (2) ME331050/1 poly-l-lactic acid (PLLA), with a thickness of 0.05 mm, and (3) BV301025/1 poly(3-hydroxybutyrate/3-hydroxyvalerate (PHB/HV), with a thickness of 0.025 mm. All plastic films were purchased from Goodfellow Cambridge, Ltd. (Huntingdon, Cambridgeshire, U.K.). In addition, a commercially available bio-based, biodegradable film made of (4) plasticized starch (PR, Bioska+, Walki Plastiroll Oy, Ylöjärvi, Finland) was used, with a thickness of 0.02–0.025 mm.

### *In Situ* Incubations

All materials were cut into 50 mm diameter circles and placed in the sample holders modified from polystyrene analyslide Petri dishes (Pall Corporation, Port Washington, NY, U.S.A.; Supplementary Figure 1 of the Supporting Information). Both sides of the analyslide dish were cut open as wide as possible with an electric hot-wire cutter to allow free flow of seawater. Experimental pieces were closed in dishes, and their lids were secured in place with small cable ties. For each material, three to four replicates were prepared for weight-attrition measurements and three replicates were prepared for bacterial community analysis. Individual sample holders were placed in the clear polystyrene boxes with sides cut open, so that all samples were in an upright position 2 cm apart inside the box (Supplementary Figure 1 of the Supporting Information). The boxes were attached to frames constructed of 50 mm diameter polyvinyl chloride (PVC) pipes with dimensions of 1.2 × 1.2 m. The frames were anchored in June 2018 in an upright position (to prevent sedimentation onto the samples) at a depth of approximately 8 m, 1 m above the bottom in a sheltered coastal location (59° 50′ 33.9″ N, 23° 15′ 40.9″ E) on the southwest coast of Finland. The samplings were conducted by removing the entire frame after 6 and 12 months of incubation. During the incubation, the measured salinity range was 4.29–6.76, the temperature was from −0.37 to 25.12 °C, the pH was 7.64–9.10, and the dissolved oxygen concentration was 6.05–17.82 mg L^–1^ at a nearby continuous Tvärminne Zoological Station MONICOAST monitoring site at 4 m depth and approximately 600 m distance from the study site (www.helsinki.fi/monicoast). A complete time series plot of environmental data is included in Supplementary Figure 2 of the Supporting Information.

### Weight Attrition

**Figure 1:**
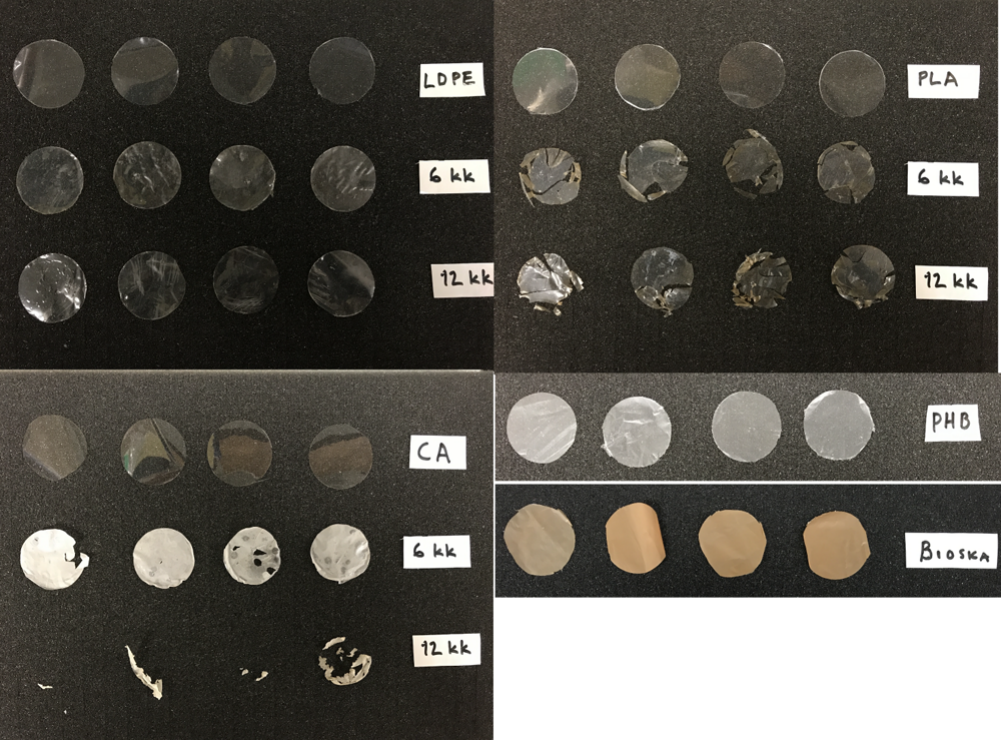
Picture of the cleaned plastic materials used in the *in situ* incubations at the beginning of the experiment, after 6 months (6 kk) and 12 months (12 kk). LDPE, low-density polyethylene; CA, cellulose acetate; PLLA, poly-l-lactic acid; PHB/HV, poly(3-hydroxybutyrate/3-hydroxyvalerate; and Bioska, plasticized starch (PR).

After sampling, the replicate samples were stored in a freezer (−20 °C) before processing. The samples were carefully cleaned of any epiphytic growth and dried for 48 h at 60 °C in a drying cabinet. After drying, the replicate samples were individually weighed with an analytical balance (Sartorius AG, Göttingen, Germany) to an accuracy of 1 mg. Weight attrition was calculated from the averages of all replicates for each material against the average weight of the corresponding number of untreated new material replicates. Surface erosion of the materials was qualitatively examined with fluorescence stereomicroscopy, using a Leica MZ 7.5 stereomicroscope (green-light excitation, magnification of 0.63–5.0×, Leica Camera AG, Wetzlar, Germany). The results corresponded to the visual observation of materials after incubation (Figure 1). In PE and PLLA, appearing clear after incubation, no surface erosion was seen in microscopy, whereas in rapidly degrading PR and PHB/HV as well as CA (opaque after incubation), the surface was clearly eroded. Of the materials used, the PLLA shrank and fragmented during incubation, while PHB/V and PR were mostly degraded during the first 6 months of incubation. To ensure that weight attrition was not due to fragmentation and subsequent loss of the detached pieces, the replicate PLLA samples after 6 and 12 months were imaged and the radius and surface area of each replicate were determined with open-source ImageJ software.^30^ From image analysis, we deduced that shrinkage and not fragmentation of PLLA was the cause of the loss of surface area during incubation. Although PLLA cracked, the fragments were not detached from the frames and lost during incubation. For rapidly degrading materials (PHB/V and PR), weight attrition by fragmentation was controlled in a separate experiment, in which the materials were encased in fine (mesh size of 20 μm) stainless-steel mesh and incubated in sample frames at the study location for 8 months from February 2019 to October 2019. The materials were also degraded at a comparable rate inside the fine mesh with no substantial fragmentation.

### Biodegradation Experiments

To determine the biodegradation to CO_2_, a series of three separate laboratory experiments were carried out according to standard ASTM D6691-17^31^ with seawater from the study site. We used a Wissenschaftlich Technische Werkstätten (WTW) OxiTop control 12 respirometric biodegradation measurement system, which records biological oxygen demand by pressure difference from the absorption of CO_2_ evolved during incubation into sodium hydroxide. Experimental units were 600 mL amber Duran (DWK Life Sciences, Mainz, Germany) Youtility bottles with 250 mL of natural unfiltered surface seawater collected near the study site immediately prior to initiation of the experiment. To ensure nutrient availability not limiting the degradation rate during the experiment, 100 mg L^–1^ KH_2_PO_4_ and 500 mg L^–1^ NHCl_4_ was added to each experimental bottle. Each experiment consisted of microcrystalline cellulose with an average particle size of 90 μm (Xylem Analytics, Weilheim, Germany) as a positive control, negative seawater control with no added substrate, and two finely (approximately <0.5 mm grain size) milled bioplastic materials (added in a concentration of 250 mg L^–1^) in triplicate. The materials were milled with a rotary-blade mill after cooling in liquid nitrogen to decrease their tensile strength. The experiments were run in the dark and under stirring at 15 °C for 28 days and were carried out consecutively in February (PR), April (PLLA and PHB/HV), and May (CA and LDPE) 2019. The biological oxygen demand recorded automatically by WTW OxiTop from pressure difference was translated to biodegradation, using individually measured material carbon content values (w/w; Supplementary Table 1 of the Supporting Information) determined externally by Australian Laboratory Services ALS Czech Republic s.r.o. Prague according to the standard.^32^

### Sampling for Bacterial Community Composition

The plastics from the *in situ* incubations were collected in cryovials with sterile tweezers and subsequently transferred to −80 °C. For PHB/HV and PR, which were almost completely degraded after 6 months, the samples that had the most material left were selected to DNA extractions.

At the beginning of each biodegradation experiment, 3 × 1000 mL seawater, collected with a 5 L Limnos water sampler (Limnos oy, Turku, Finland) from the study site, was filtered onto sterile 0.22 μm membrane filters (Ø of 47 mm, Whatman GE Healthcare, Little Chalfont, Kent, U.K.). At the end of the experiment, the entire volume of 250 mL of seawater with plastics (i.e., three replicate samples) was filtered onto sterile 0.22 μm membrane filters (Ø of 47 mm, Whatman GE Healthcare, Little Chalfont, Kent, U.K.) and subsequently transferred to −80 °C.

### DNA Extraction

The plastics and membrane filters were transferred to PowerBead tubes with sterile tweezers, and DNA was extracted with a DNeasy PowerSoil kit (Qiagen, Hilden, Germany) and subsequently stored at −80 °C for further processing. Kitomes, i.e., extractions without sample material, were also performed. For sequencing, the 16S rRNA gene region V3–V4 was amplified with a two-step polymerase chain reaction (PCR), using the universal bacterial primers 341F and 785R.^33^ The PCR and Illumina MiSeq (Illumina, Inc., San Diego, CA, U.S.A.) paired-end multiplex sequencing (300 + 300 bp) were performed at the Institute of Biotechnology, University of Helsinki, Finland.^34^ In total, 7.6 million raw-read pairs were obtained with the Illumina MiSeq platform. Primer removal was performed with Cutadapt (settings -m 1\\O 15 -e 0.2, V 2.7^35^), and the reads were merged and processed according to the DADA2 pipeline (DADA2, version 1.8^36^) with filterAndTrim maxEE = 2. After filtering and trimming, a total of 5.5 million read pairs remained, of which 2.5 million were merged and 2.2 million were non-chimaeric and used for further analyses. Taxonomic classification of the amplicon sequence variants (ASVs) was performed with DADA2 default parameters (minBoot = 50), using Silva for DADA2 (version 132^37,38^). Before the statistical analyses, the chloroplast and mitochondria sequences were removed, ending up with 3787 ASVs in the degradation experiments and 7965 ASVs in the *in situ* incubations. The raw reads were deposited in the Sequence Read Archive of the National Centre for Biotechnology Information, under BioProject accession number PRJNA849282.

### Statistical Analysis

Pairwise comparison between the conventional plastic (LDPE) and the bioplastic materials (PLLA, CA, PHB/HV, and PR) was performed to investigate whether certain bacterial genera were enriched on bioplastic materials. DESeq2 (version 1.28.1^39^) with default parameters was used to analyze the differentially abundant taxa between the bacterial communities, using an adjusted *p* value of 0.05 as a cutoff.

The figures were drawn with R (4.0.2^40^) using ggplot2 (version 3.3.5^41^) and with phyloseq (version 1.32.0^42^). Principal coordinate analysis (PCoA) was performed on a Bray–Curtis dissimilarity matrix derived from square-root-transformed values. All scripts for processing sequence data are available in the Supporting Information.

## Results and Discussion

### *In Situ* Incubations

**Figure 2:**
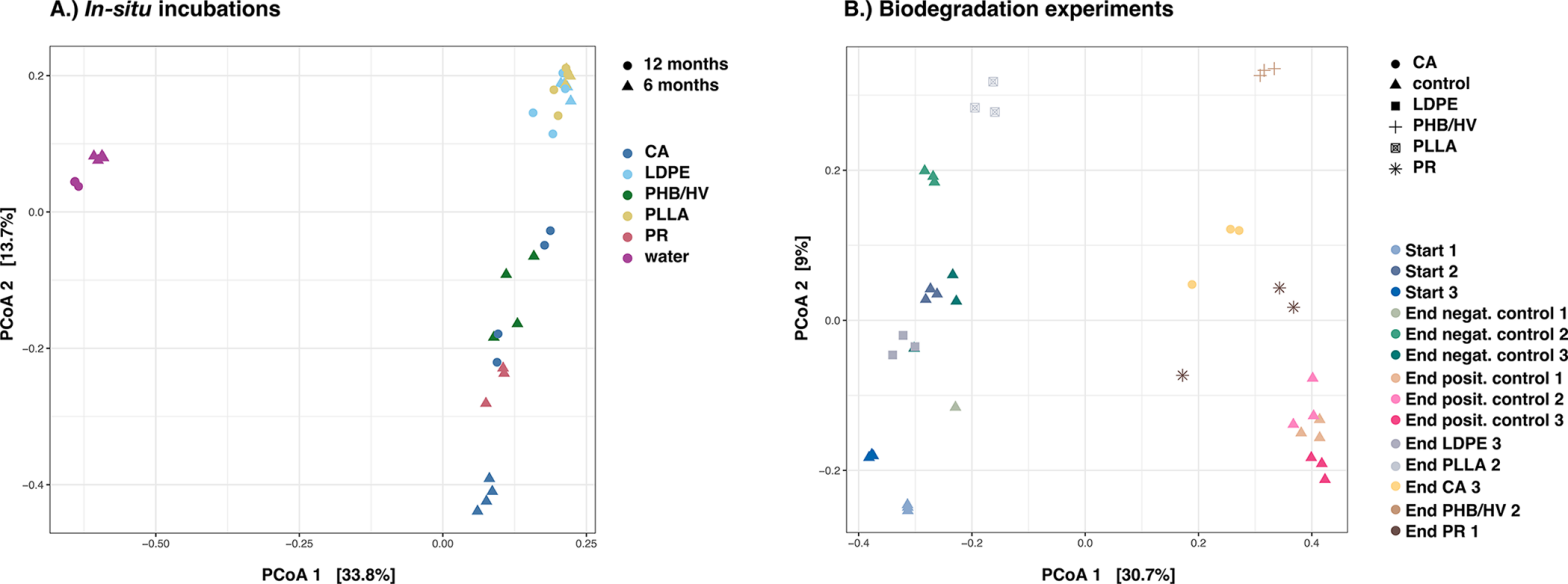
PCoA based on the square-root-transformed Bray–Curtis dissimilarity matrix of the bacterial 16S rRNA gene sequences showing bacterial community dynamics on different plastic types (LDPE, low-density polyethylene; CA, cellulose acetate; PLLA, poly-l-lactic acid; PHB/HV, poly(3-hydroxybutyrate/3-hydroxyvalerate; and PR, plasticized starch) in (A) *in situ* incubations and (B) biodegradation experiments. The numbers 1–3 indicate the experimental batch.

**Figure 3:**
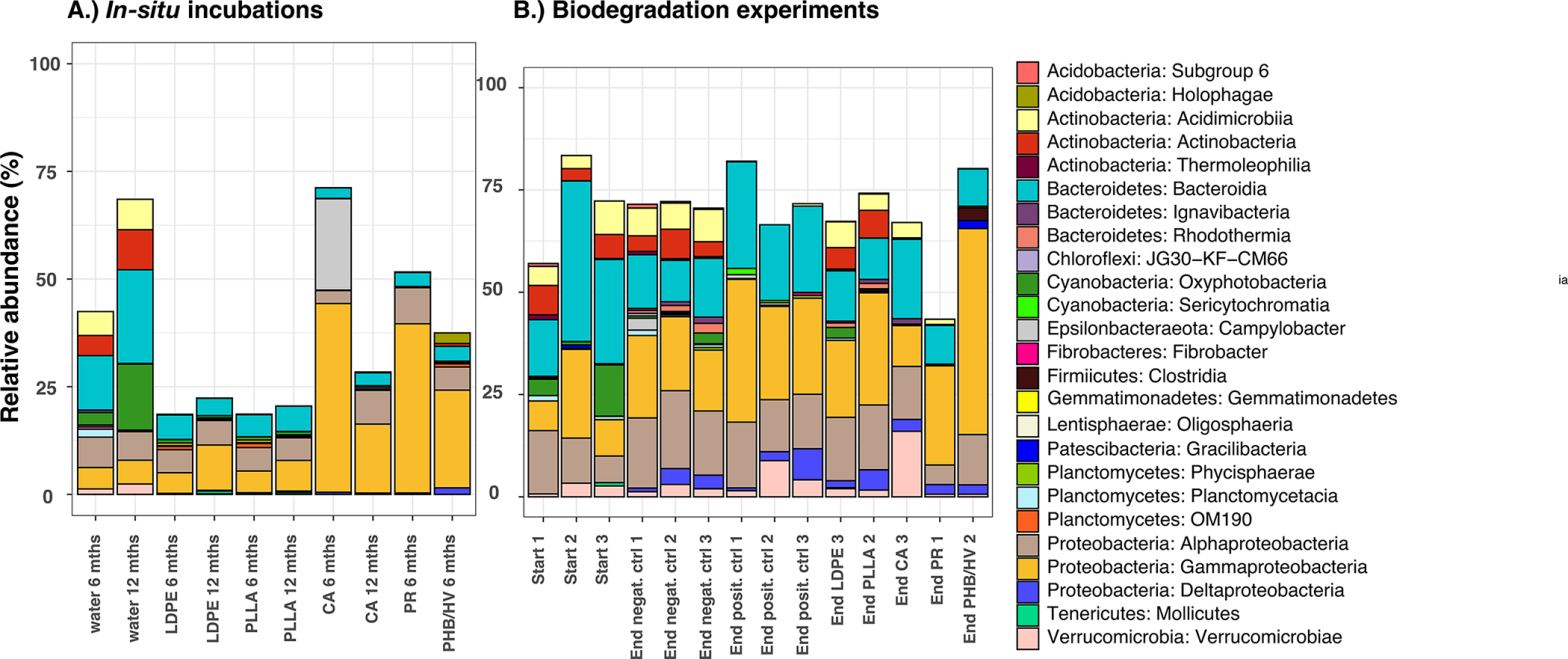
Class-level bacterial diversity of the 16S rRNA gene sequences [∼450 base pairs (bp)] representing >0.5% of all amplicon sequence variants (ASVs) on different plastic types (LDPE, low-density polyethylene; CA, cellulose acetate; PLLA, poly-l-lactic acid; PHB/HV, poly(3-hydroxybutyrate/3-hydroxyvalerate; and PR, plasticized starch) in (A) *in situ* and (B) biodegradation experiments. mths, months; ctrl, control.

Four different bioplastic materials (PHB/HV, PR, PLLA, and CA) and conventional LDPE as a reference were incubated for 1 year to determine the weight attrition and bacterial community composition on biodegradable plastics in a brackish marine environment. Among the plastic samples, the reference material (LDPE) and the biodegradable PLLA clustered together both after 6 and 12 months (Figure 2) and were dominated by Alphaproteobacteria, Gammaproteobacteria, and Bacteroidia at both time points (Figure 3). The results indicate that the bacterial communities on LDPE and PLLA were matured and stable, which was expected after such a long incubation time (reviewed in ref (20)). In line with these results, neither LDPE nor PLLA showed surface erosion in light microscopic examination attributable to biodegradation. However, PLLA shrank and fragmented during the 12 month incubation, with visible fragmentation taking place already after 6 months (Figure 1). Nevertheless, this fragmentation is likely to lead only to the formation of smaller plastic particles and, hence, does not alleviate the plastic pollution load. Thus, on the basis of our results, PLLA is not biodegradable in an open marine environment, as already suggested in previous studies.^23−25^

In contrast to PLLA, the PHB/HV and PR foils were almost completely degraded after 6 months (weight attrition of 99.9 and 99.7%, respectively; Supplementary Table 1 of the Supporting Information), whereas CA lost 80% of its weight after 12 months (Figure 1). However, the slower degradation of CA may also have resulted from the CA film being thicker (0.05 mm) than the PHB/HV (0.025 mm) and varying PR (0.02—0.025 mm) films. This was also reflected in the bacterial community composition, because PHB/HV and PR after 6 months clustered together with the CA samples after 12 months (Figure 2), indicating that the community development on PHB/HV and PR was more similar and faster than that on CA. In comparison to LDPE and PLLA, CA, PR, and PHB/HV showed high percentages of Gammaproteobacteria after 6 months (LDPE, 4.8%; PLLA, 5%; CA, 43.8%; PR, 39.3%; and PHB/HV, 22.7%; Figure 3). Gammaproteobacteria are common primary colonizers on plastic materials.^21,44−46^ However, on the basis of our results, it seems that they are also abundant after the maturation phase. This may result from the material properties or associated biofilms, which may provide continuous substrate resources for the bacterial community, thus keeping the bacterial community in the active growth phase. Nevertheless, because the weight attrition of these plastics was clearly detected, we deem it plausible that the plastics served as a carbon source for these bacterial communities and caused the differences in community composition. There is also previous evidence for possible biodegradation of PHB in marine environments; many natural marine bacteria are known to possess genes for PHB biodegradation.^21,22,24,27,47^

**Figure 4:**
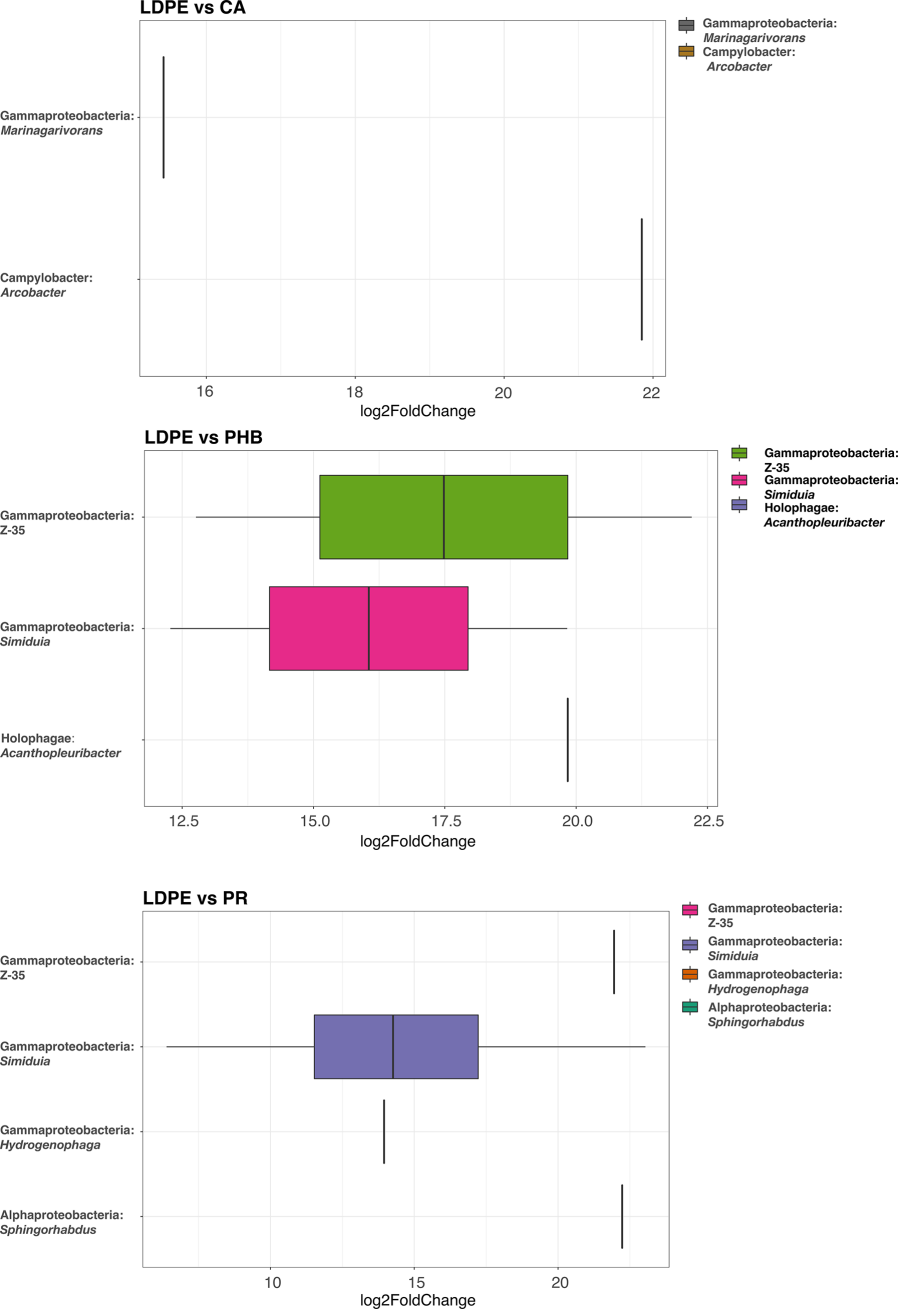
Differentially abundant 16S rRNA genes showing bacterial genera enriched (representing >0.5% of the total community) on (A) CA = cellulose acetate, (B) PHB = poly(3-hydroxybutyrate/3-hydroxyvalerate, and (C) PR = plasticized starch in *in situ* incubations, in the Baltic Sea.

When the bacterial community composition was investigated in greater detail, certain genera were significantly enriched on PHB/HV, PR, and CA bioplastics compared to the reference material LDPE (*p*_adj_ > 0.05; relative abundance over 0.5%), according to differential abundance analysis (Figure 4). On CA, *Arcobacter* (Epsilonproteobacteria) and *Marinagarivorans* (Gammaproteobacteria) were significantly enriched (LDPE, 0%; CA, 21.2 and 20.1%, respectively); on PR, *Sphingorhabdus* (Alphaproteobacteria; LDPE, 0.1%; PR, 3.4%), *Simiduia* (Gammaproteobacteria; LDPE, 0%; PR, 25%), Z-35 (Gammaproteobacteria; LDPE, 0%; PR, 2.8%), and *Hydrogenophaga* (Gammaproteobacteria; LDPE, 0%; PR, 6.2%) were significantly enriched; and on PHB, *Simiduia* (Gammaproteobacteria; LDPE, 0%; PHB, 7%), Z-35 (Gammaproteobacteria; LDPE, 0%; PHB, 7%); and *Acanthopleuribacter* (Holophaga; PE, 0.04%; PHB, 2.5%) were significantly enriched (Figure 4 and Supplementary Figure 3 of the Supporting Information). Many of these enriched genera represent bacterial taxa putatively capable of consuming complex carbon substrates, and/or they have also been detected on plastics in previous studies.^43,44,48,49^ Despite the brackish nature of the Baltic Sea, common plastisphere genera were detected. *Arcobacter* (21.2%), which was one of the most abundant genera on CA after 6 months (Figure 3 and Supplementary Figure 3 of the Supporting Information), is a common plastisphere genus, also detected previously on plastics in sediments.^43,44^*Arcobacter* spp. are also capable of using complex substrates, such as acetate.^50^ Moreover, *Hydrogenophaga* is listed as a core genus on plastics, and *Sphingorhabdus* is a member of the family Sphingomonadaceae, which is often associated with plastic polymers (reviewed in ref (9)).

On PE and PLLA, the most abundant genera after 6 months were *Rhodobacter* (Alphaproteobacteria, 1.1% on both) and *Defluviimonas* (Alphaproteobacteria, 0.9% on both), and after 12 months, the most abundant genera were *Aeromonas* (Gammaproteobacteria; PE, 4.2%; PLLA, 0%), *Paraglaciecola* (Gammaproteobacteria; PE, 0.7%; PLLA, 1.4%), and *Rhodobacter* (Alphaproteobacteria; PE, 1.2%; PLLA, 1.3%; Supplementary Figure 3 of the Supporting Information). Bacteria belonging to family Rhodobacteraceae have also been detected on PE previously.^9^ In addition, both *Defluviimonas* spp. and *Aeromonas* are known of their ability to degrade polycyclic aromatic hydrocarbons.^51,52^ Nevertheless, despite the potential degrader bacteria on PE and PLLA, their intactness indicates that degradation of these plastics in brackish marine environments in a year is negligible.

In all, the results indicate that the weight attrition of different biodegradable bioplastic materials varies greatly in Baltic Sea water: PLLA is very resistant to degradation, whereas PHB/HV and PR both degrade rapidly in seawater. CA degrades in the open marine environment as well, but the process is slower than it is with PHB/HV and PR under these conditions.

### Biodegradation Experiments

Three 1 month long incubations with milled plastic materials were carried out to measure the actual biodegradation (i.e., full conversion to CO_2_ under aerobic conditions) of plastic materials in brackish Baltic Sea water. As in the *in situ* incubations, the bacterial communities on LDPE and PLLA were very similar, resembling those in the initial water samples (Figure 3 and Supplementary Figure 3 of the Supporting Information). The classes Bacteroidia, Alphaproteobacteria, Gammaproteobacteria, and Actinobacteria predominated in the initial water samples, negative controls, and reference material LDPE and bioplastic PLLA, and these samples clustered along the *y* axis, separately from the positive controls, PHB/HV, CA, and PR (*y* axis explaining 9% of the variation; Figures 2 and 3 and Supplementary Figure 3 of the Supporting Information). On the basis of the CO_2_ flux measurements, neither LDPE nor PLLA showed any signs of biodegradation during the 1 month incubation, indicating that these materials were not biodegradable under these conditions.

CA, PR, and PHB/HV were dominated by the classes Alphaproteobacteria, Gammaproteobacteria, Bacteroidia, and Verrucomicrobiae (Figure 3). On PR and PHB/HV, Gammaproteobacteria predominated the communities (24.2 and 50.3%, respectively; Figure 3). In comparison to the negative controls, the relative abundance of Gammaproteobacteria was almost 3 times higher on PHB/HV, whereas on PR, the relative abundance was only 4% higher by the end of the experiment. In the PR, there were various Gammaproteobacteria with similar percentages (with the top four being *Hydrogenophaga* at 3%, *Paraglaciecola* at 2.7%, *Acidovorax* at 2.3%, and *Pseudomonas* at 2%), whereas on PHB/HV, the genus *Pseudomonas* clearly predominated in the community (30.7%), followed by *Acidovorax* (5%). The predominance of Gammaproteobacteria is not surprising, because Gammaproteobacteria are known to be the primary colonizers on plastics.^21,46^ In addition, *Pseudomonas* spp. are well-known for their poly-3-hydroxyalkanoic acid (PHA)-producing/-degrading capability^53^ as well as other polymer- and petroleum-degrading capabilities.^54,55^ Active bacterial communities on PHB have also been observed in previous studies, indicating potential biodegradation of PHB in marine environments.^21,24,56^ However, on the genus level, *Flavobacterium* was the most abundant taxa on PR (4.2%; negative control, 0.7%; Supplementary Figure 3 of the Supporting Information). Also, *Flavobacterium* is a plastisphere taxa,^55^ and Bacteroidetes are known for their ability to degrade complex substrates, e.g., in algal blooms.^57^ In contrast to PHB and PR, on CA, verrumicrobial abundance increased from 2.8% in the beginning of the experiment to 16% in CA treatment (Figure 3) during the course of the experiment, with *Prosthecobacter* being the most dominant genera (13.4%; Supplementary Figure 3 of the Supporting Information). *Prosthecobacter* has also been detected on plastics previously.^58,59^

**Figure 5:**
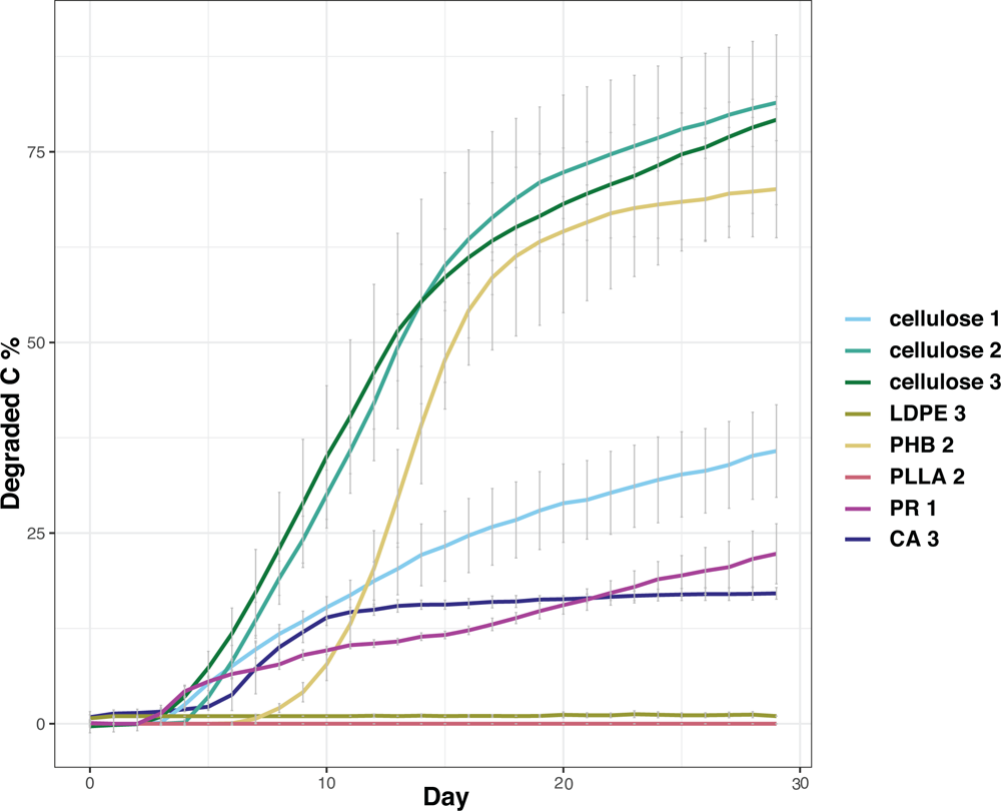
Loss of carbon as a percentage in 28 day laboratory incubations. LDPE, low-density polyethylene; CA, cellulose acetate; PLLA, poly-l-lactic acid; PHB/HV, poly(3-hydroxybutyrate/3-hydroxyvalerate; and PR, plasticized starch. Cellulose was used as a positive control. The numbers 1–3 indicate the experimental batch. Seawater controls were 0 in all three experiments and, therefore, not presented in the figure.

PHB/HV biodegraded up to 70% by the end of the experiment, whereas CA biodegraded only about 15% during the first 10 days, after which the biodegradation rate slowed (Figure 5). CA biodegradation is a more complex process than cellulose biodegradation, because esterases are needed to first break the acetyl group.^60^ The degree of substitutions (DS) determines how easily CA can be biodegraded; the higher levels of acetyl substitutes decrease the biodegradation rates, and therefore, all CAs cannot be considered equally degradable.^60^ In this study, biodegradable CA with low DS was used, likely increasing the biodegradation rate. However, the rapidly degraded part of CA may also be the biodegradation of additives, because they can form 20–30% of the material mass and certain additives enhance CA biodegradability in compost.^20,61^ However, because we are not aware which additives were used, we are not able to assess their effect.

PR also degraded only up to 20%, but because the positive control (cellulose) degraded much more slowly (∼40% less; Figure 5) than in the other experiments, it is likely associated with the winter conditions in February when the water for this experiment was collected. However, because the nutrients were added in excess at the beginning of the experiment, the temperature was constant in all experiments, and there were no notable differences in the bacterial community composition, the reason was likely associated with the slower bacterial process in winter with a higher carbon demand to acclimate. Previous evidence has also shown that the environmental conditions and the season may affect the potential community composition and, therefore, also the degradation potential.^17,18,62^ There may also be other potential plastic-degrading organisms, such as fungi, missing from the winter community or bacteria and/or archaea not detected as a result of the primer bias. Chytridiomycetes have been detected on plastic biofilms in several studies in both marine^63,64^ and fresh water;^65^ however, their role in plastic degradation remains elusive. Nevertheless, because we sequenced only the 16S rRNA gene, we cannot verify the role of fungi with our data.

Also, on PE and PLLA, a slight increase on gammaproteobacterial abundance was observed. On the genus level, the most abundant genera on PE were *Paraperlucidibaca* (Gammaproteobacteria, 4.6%; negative control, 0.5%) and hgcI clade (Actinobacteria, 4%; negative control, 3.4%), whereas on PLLA, *Sphingorhabdus* (Alphaproteobacteria, 7.9%; negative control, 7%) and *Simplicispira* (Gammaproteobacteria, 4.3%; negative control, 0%; Supplementary Figure 3 of the Supporting Information) were the most abundant genera. *Paraperlucidibaca* is potentially a cold-adapted, hydrocarbonoclastic bacteria.^66^ However, because no biodegradation was detected in either PLLA or PE, their role in degradation remains speculative.

Degradation of bioplastic materials in marine environments is complex because it is a combination of several abiotic and biotic factors, such as salinity, temperature, radiation, and mechanical processes, as well as the composition of the microbial community (reviewed, e.g., in ref (9)). For example, salinity decreases degradability,^26^ whereas a higher temperature and light increase it.^26,67^ In the brackish Baltic Sea, water microbial communities are a combination of marine and freshwater species with high similarity to other brackish environments, such as the Chesapeake Bay^68^, and, therefore, not directly comparable to marine bacterial communities. However, the bioplastic biofilms in this study harbored multiple of the same bacterial genera as their marine counterparts, yet distinguishing these abiotic and biotic factors and understanding the possible effects of brackish microbial communities to bioplastic degradation requires further investigations.

Nevertheless, the weight attrition together with the clear shifts in bacterial community composition in both biodegradation experiments and *in situ* incubations (i.e., predominance of Gammaproteobacteria) indicate that these bacteria are putatively degrading PHB/HV, CA, and PR. Although the general view is that marine microbes play a negligible role in the degradation of plastics,^9^ on the basis of our study, it appears to apply more to conventional plastics, whereas some bioplastic materials seem to be more prone to biodegradation. Thus, certain bioplastics, such as PHB/HV, could be a better option for applications that are at a high risk of ending up in marine environments. However, despite their degradability, it should be kept in mind that, even though bioplastics will degrade in the marine environment, their degradation products may affect marine food webs or release harmful degradation products.^13,55^

To summarize, our aim was to determine the true biodegradation of various bioplastic materials (PLLA, PHB/HV, PR, and CA) in the brackish Baltic Sea, both under open environment conditions as well as in controlled laboratory experiments. Baltic Sea water always seems to have a potential degrader community present; however, the rate of degradation is slower during winter months. The effect of the inoculum in standardized bioplastic degradation testing in seawater may need to be further addressed before reliable testing standards across future marine environments and seasons can be established. The results show that PHB/HV and PR are especially quickly degraded in seawater and that CA also has higher degradation potential than LDPE, even though the rate is slower than for PHB/HV and PR. Interestingly, PLLA, which is very commonly used in various disposable items, such as paper cups,^69^ showed no signs of degradation, either under the experimental conditions or in the environment. Our results emphasize the need to carefully consider the selection of biodegradable material in applications with the risk of ending up in the marine environment.

## Supporting Information Available

Tables and figures showing detailed pictures and description of the carbon content of materials and weight loss of *in situ* incubations (Supplementary Table 1), close-up of the sampling rack (Supplementary Figure 1), time series plots of background environmental data for *in situ* incubations (Supplementary Figure 2), and heatmaps showing genus-level bacterial diversity of 16S rRNA gene sequences (Supplementary Figure 3) and R-scripts (PDF)

## Abbreviations

LDPE: low-density polyethylene
CA: cellulose acetate
PLLA: poly-l-lactic acid
PHB/HV: poly(3-hydroxybutyrate/3-hydroxyvalerate
PR: plasticized starch
