# Supplementary material for: Degradation Rates and Bacterial Community Compositions Vary among Commonly Used Bioplastic Materials in a Brackish Marine Environment
Source: Environ Sci Technol. 2022 Oct 21;56(22):15760–9. doi: 10.1021/acs.est.2c06280 (PMC9671047; doi:10.1021/acs.est.2c06280)
Supplement: Supplementary file 1 — es2c06280_si_002.pdf [file es2c06280_si_002.pdf]

Supporting information for article:

" Degradation rates and bacterial community compositions vary among commonly used bioplastic materials in brackish, marine environment"

Environmental Science & Technology

Eeva L. Eronen-Rasimus*[1,2], Pinja P. Näkki[2] and Hermanni P. Kaartokallio[2]

[1]University of Helsinki, Department of Microbiology, Viikinkaari 9, 00790 Helsinki, Finland

[2] Finnish Environment Institute, Marine Research Centre, Agnes Sjöbergin katu 2, 00790 Helsinki, Finland

Author Information:

*Eeva Eronen-Rasimus,

work was done in: Finnish Environment Institute, Marine Research Centre, Agnes Sjöbergin katu 2, 00790 Helsinki, Finland

Current address: University of Helsinki, Department of Microbiology, Viikinkaari 9, 00790 Helsinki, Finland

eeva.eronen-rasimus@helsinki.fi

https://orcid.org/0000-0002-1749-0793

Pinja P. Näkki

Finnish Environment Institute, Marine Research Centre, Agnes Sjöbergin katu 2, 00790 Helsinki, Finland

pinja.nakki@syke.fi

https://orcid.org/0000-0003-2499-4751

Hermanni P. Kaartokallio

Finnish Environment Institute, Marine Research Centre, Agnes Sjöbergin katu 2, 00790 Helsinki, Finland

hermanni.kaartokallio@syke.fi

https://orcid.org/0000-0002-3650-4628

Supporting information showing tables and figures on *in-situ* incubation racks, background environmental parameters in *in-situ* incubations and carbon content & weight loss in *in-situ* incubations as well as genus level bacterial community composition in both *in-situ* incubations and biodegradation experiments.

Suppl. table 1: Carbon content of materials and weight loss of in *in-situ* incubations.

MATERIAL	CARBON CONTENT	EXPERIMENTAL PIECE WEIGHT			WEIGHT LOSS	
		before incubation	after 6 months	after 12 months	after 6 months	after 12 months
	%, mean±SE	*mg, mean±SE*			*%, mean*	
**LDPE**	88.9 ± 0.6 ,n=3	77.1 ± 1.8 ,n=6	80.0 ± 3.0 ,n=4	75.7 ± 4.1 ,n=4	-3.8	1.8
**CA**	52.6 ± 0.7 ,n=3	121.4 ± 1.0 ,n=6	55.2 ± 3.2 ,n=4	24.7 ± 18.3 ,n=4	54.5	79.7
**PLLA**	50.8 ± 0.2 ,n=3	113.2 ± 1.7 ,n=6	99.2 ± 4.2 ,n=4	101.7 ± 4.3 ,n=4	12.4	10.2
**PHB/HV**	58.9 ± 0.0 ,n=1	50.5 ± 1.6 ,n=6	N/A	N/A	100	100
**PR**	55.8 ± 0.2 ,n=3	68.3 ± 9.6 ,n=6	N/A	N/A	100	100

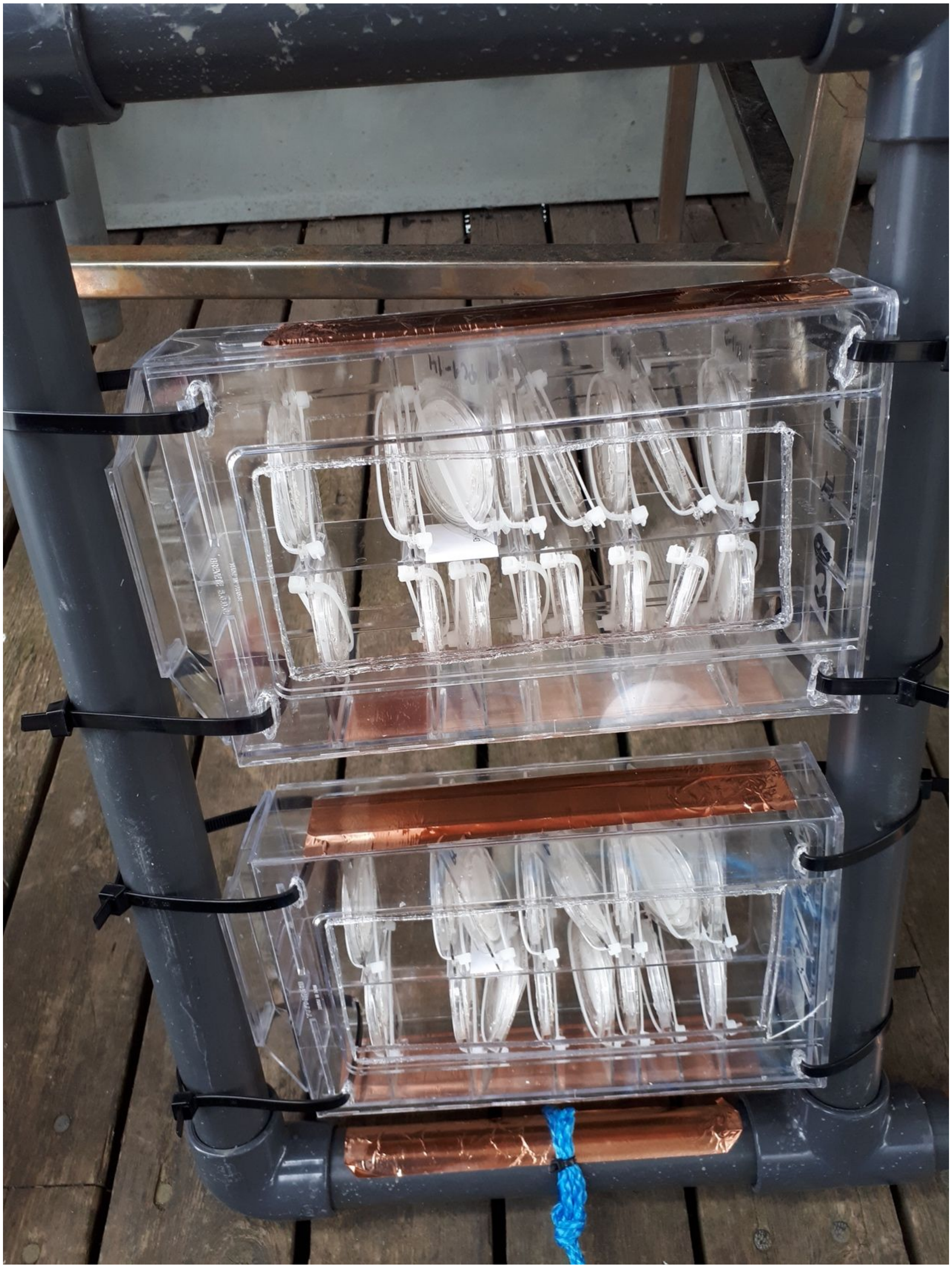

Supplementary Figure 1

Picture of a sample holder in *in-situ* incubations. Samples inside sample holders placed in the polystyrene box and attached into incubation frame. Two individual identical incubation frames were used, with one of them recovered after 6 or 12 months.

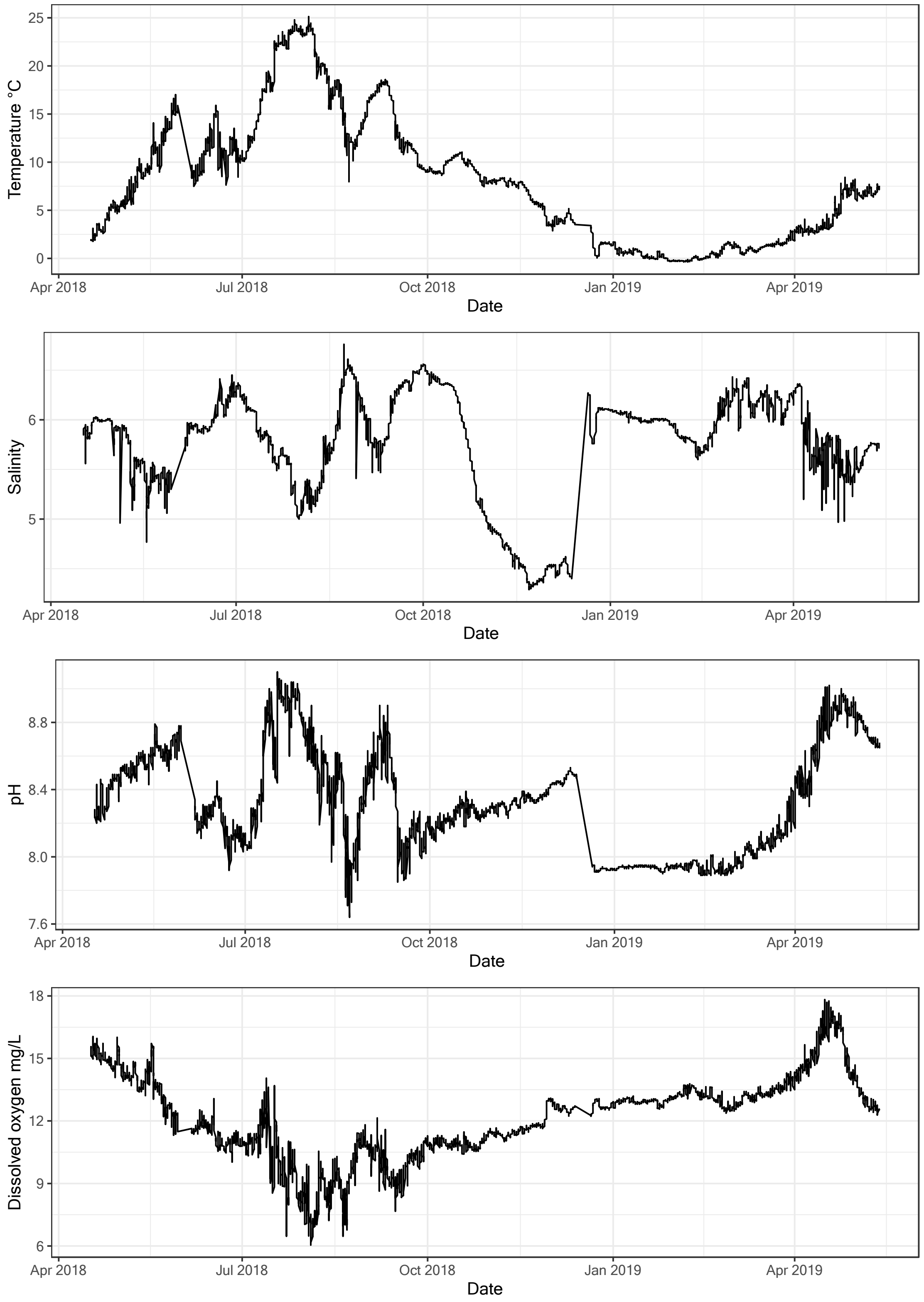

Supplementary Figure 2

Time series plots of background enviromental data for *in-situ* incubations including the entire incubation time from June 2018 to June 2019. The data is from University of Helsinki MONICOAST long-term continuous monitoring (www.helsinki.fi/monicoast) site.

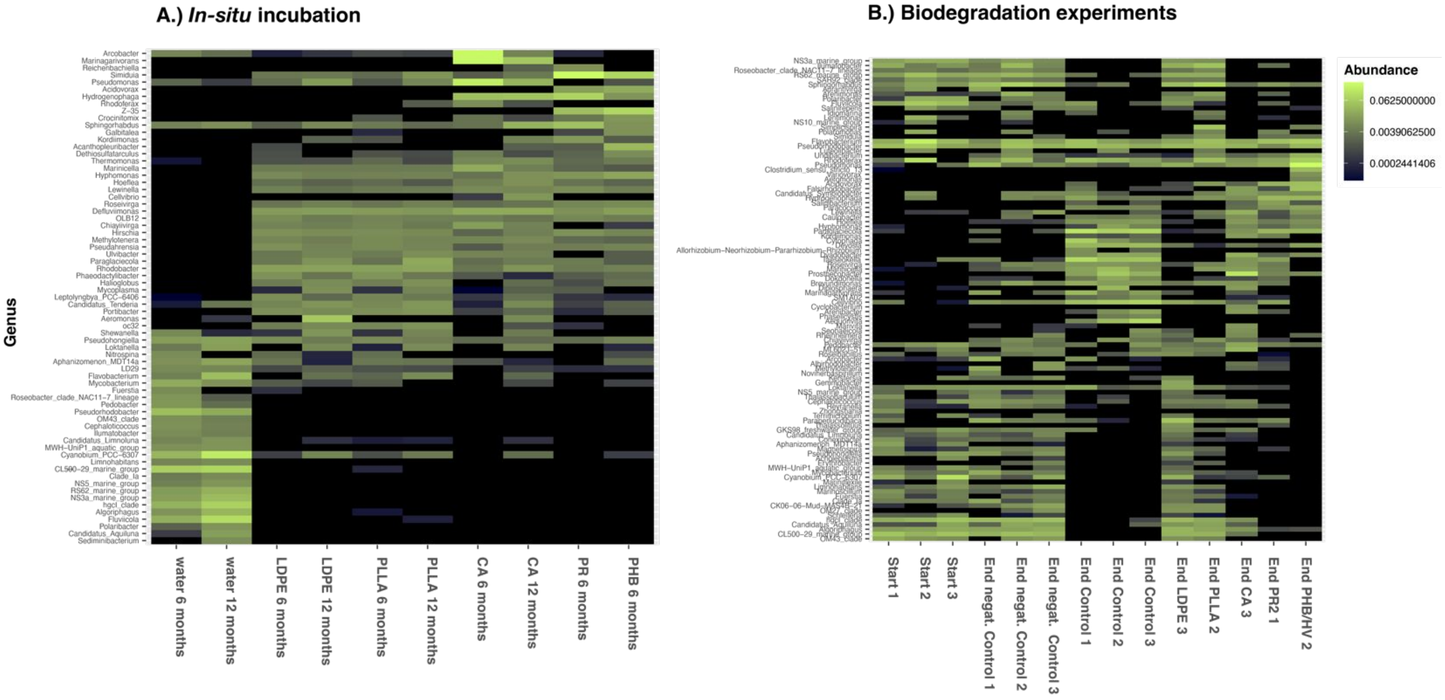

Supplementary Figure 3

Heatmap showing genus-level bacterial diversity of 16S ribosomal RNA (rRNA) gene sequences (~450 base pairs bp) representing > 0.5% of all amplicon sequence variants (ASVs) on different plastic types: LDPE = low-density polyethylene, CA = cellulose acetate, PLLA = poly-L-lactic acid, PHB/HV = poly(3-hydroxybutyrate/3-hydroxyvalerate and PS = plasticized starch in A.) *In-situ* incubations and B.) Biodegradation experiments.

R-scripts UBINAM

Eeva Eronen-Rasimus

8/1/2022

Load libraries

```
library(ggplot2)
packageVersion("ggplot2")
# '3.3.5'
library(phyloseq)
packageVersion("phyloseq")
# '1.32.0'
library(ggthemes)
packageVersion("ggthemes")
# '4.2.4'
```

Bring data in and make phyloseq object

```
# Bring in ASV table
ASV_TABLE_UBI <- read.table("UBINAM_V4_DADA2_ASVtable_maxee2_290220ed.txt", header=TRUE, row.names=1, dec=".")

# Bring in TAX table
TAX_TABLE_UBI <- read.table("UBINAM_V4_DADA2_TAXtable_maxee2_290220.txt", row.names=1)
colnames(TAX_TABLE_UBI) <- c("Domain", "Phylum", "Class", "Order", "Family", "Genus")

# Bring in metadata
ENV_TABLE_UBI <- read.table("UBINAM_meta_ed.txt", sep="\t", header=TRUE, row.names=1)

# Make phyloseq object
ASV_UBI <- otu_table(ASV_TABLE_UBI, taxa_are_rows=TRUE)
TAX_UBI <- tax_table(as.matrix(TAX_TABLE_UBI))
ENV_UBI<-sample_data(ENV_TABLE_UBI)
physeq_UBI <- phyloseq(ASV_UBI, TAX_UBI, ENV_UBI)
```

Remove Chl-a and mitochondrial sequences

```
Physeq_UBI_noChl <- subset_taxa(physeq_UBI, (Order!="Chloroplast") | is.na(Order))
Physeq_UBI_noChlMit <- subset_taxa(Physeq_UBI_noChl, (Family!="Mitochondria") | is.na(Family))
```

Make PCoA with all samples

```
# Tehdään PCoA srt
physeq_UBI_noChlMit_sqrt = transform_sample_counts(Physeq_UBI_noChlMit, sqrt)
ord_UBI_all <- ordinate(physeq_UBI_noChlMit_sqrt, method = "PCoA", distance = "bray")
p1 <- plot_ordination(physeq_UBI_noChlMit_sqrt, ord_UBI_all, color="Material", shape="Experiment") +
  geom_point( size = 3) +
  ggtitle("All sqrt") +
  theme_bw() +
  scale_color_ptol()
p1
```

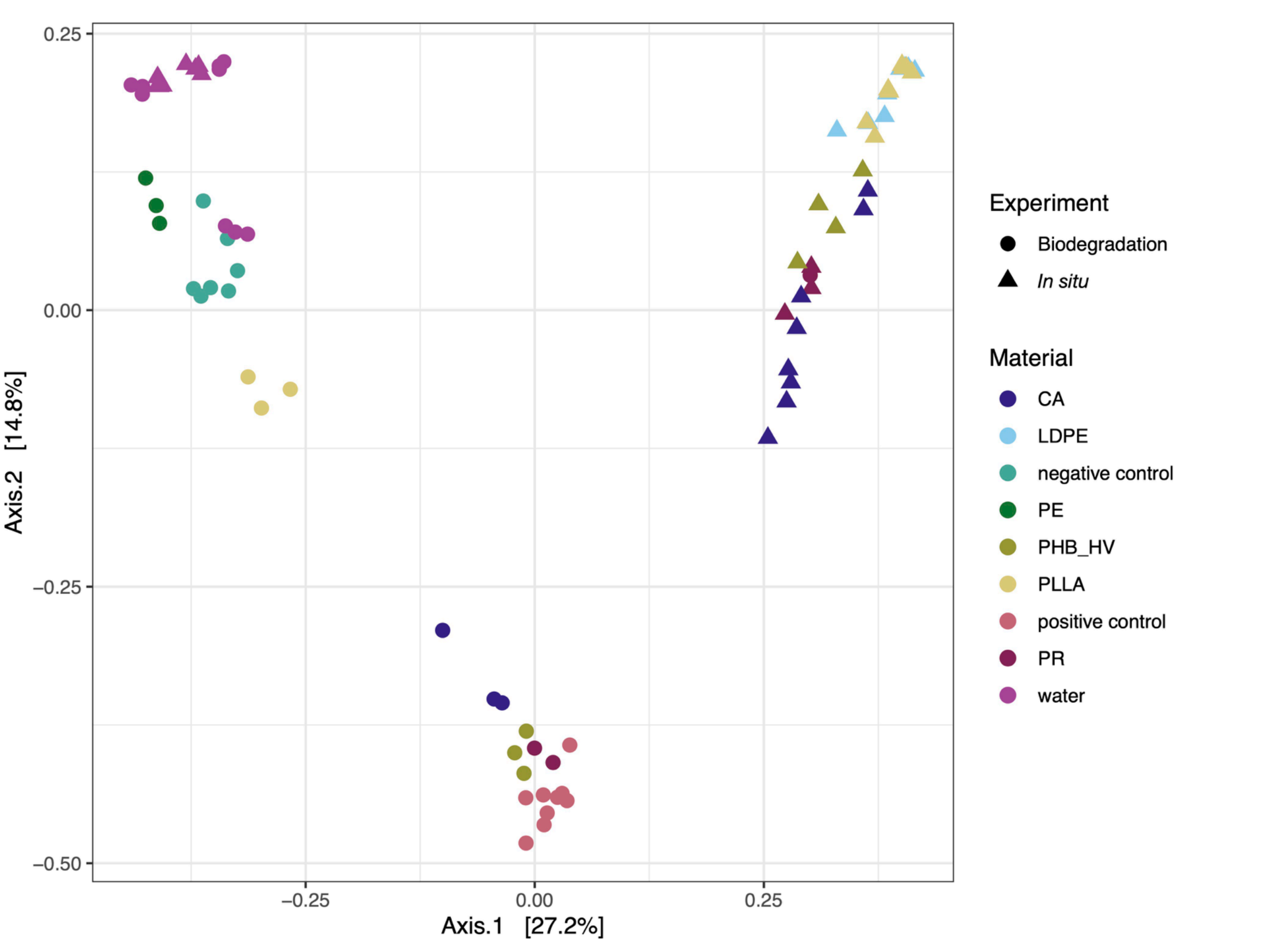

Principal coordinate analysis (PCoA) based on the square-root-transformed Bray–Curtis dissimilarity matrix of the bacterial 16S ribosomal RNA (rRNA) gene sequences showing bacterial community dynamics on different plastic types: LDPE = low-density polyethylene, CA = cellulose acetate, PLLA = poly-L-lactic acid, PHB/HV = poly(3-hydroxybutyrate/3-hydroxyvalerate and PR = plasticized starch

Table "Physeq_UBI_noChlMit" were splitted into "In-situ experiment" and "Degradation experiment". Following scripts are example how figures were drawn.

Make PCoA separately to "In-situ experiment" and "Degradation experiment" using previous PCoA script

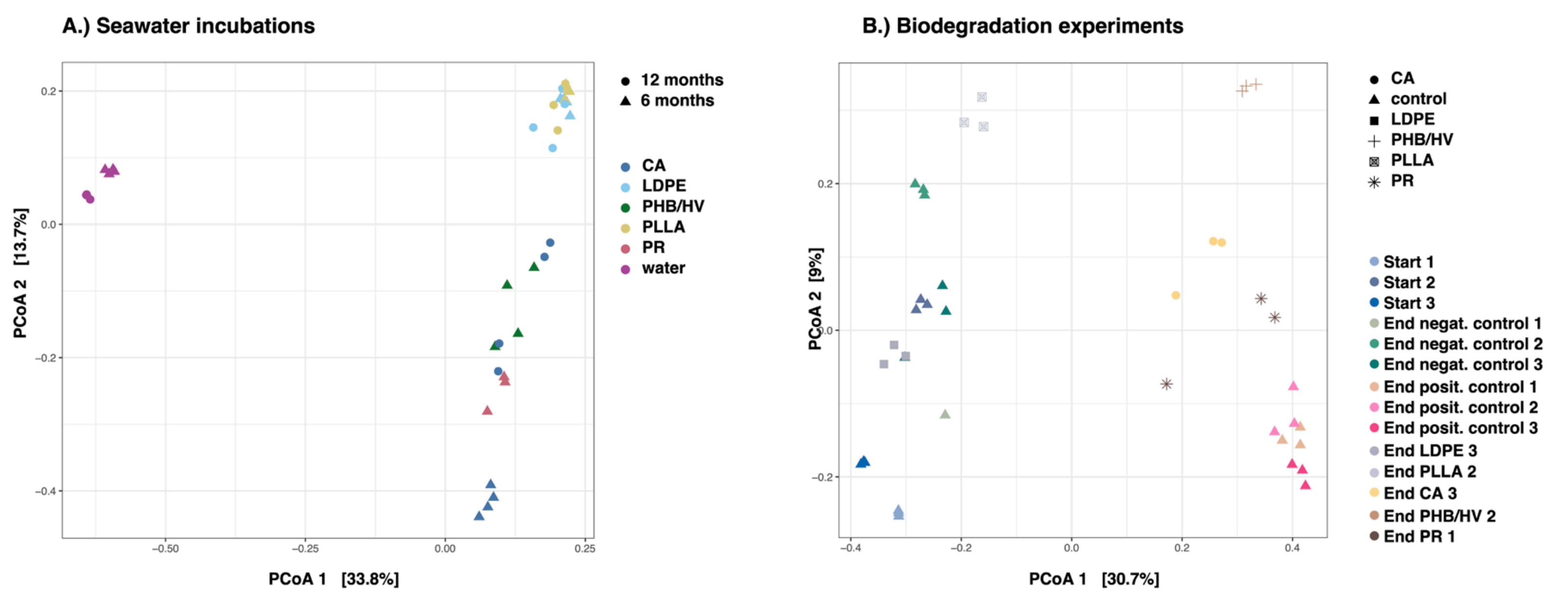

Fig.2 Principal coordinate analysis (PCoA) based on the square-root-transformed Bray–Curtis dissimilarity matrix of the bacterial 16S ribosomal RNA (rRNA) gene sequences showing bacterial community dynamics on different plastic types: LDPE = low-density polyethylene, CA = cellulose acetate, PLLA = poly-L-lactic acid, PHB/HV = poly(3-hydroxybutyrate/3-hydroxyvalerate and PR = plasticized starch in A.) In-situ incubations and B.) Biodegradation experiments. The numbers 1-3 indicate the experiment batch.

Calculate relative abundance

```
ASVs_prop_UBI <- transform_sample_counts(Physeq_UBI_noChlMit, function (x) x/sum(x))
```

Remove ASV's over 0.5%

```
ASVs_prop_UBI_05 <-filter_taxa(ASVs_prop_UBI, function(x) max(x) > .005, TRUE)
```

Select Class and Genus levels

```
ASVs_prop_UBI_05_Class <- tax_glom(ASVs_prop_UBI_05, taxrank="Class")
ASVs_prop_UBI_05_Genus <- tax_glom(ASVs_prop_HK_over_05_abun_no_out, taxrank="Genus")
```

Barplot for merged replicates (Fig.2)

```
plot_bar(ASVs_prop_UBI_05_Class, fill="Class") +
  scale_y_continuous(limits=c(0, 1)) +
  theme_bw() +
  theme(axis.text.x = element_text(angle = 90)) +
  scale_fill_manual(values=c("#a1a100", "#ffff99",  "#db2f13",  "#00c4cc",  "#774177",  "#349721",  "#cccccc",  "
#ffff00",  "#ff5ca1",  "#b9f2ff","#fe6121",   "#bda08d",  "#fabd29",  "#4c4cff",  "#0dda86",  "#ffccc3"))
```

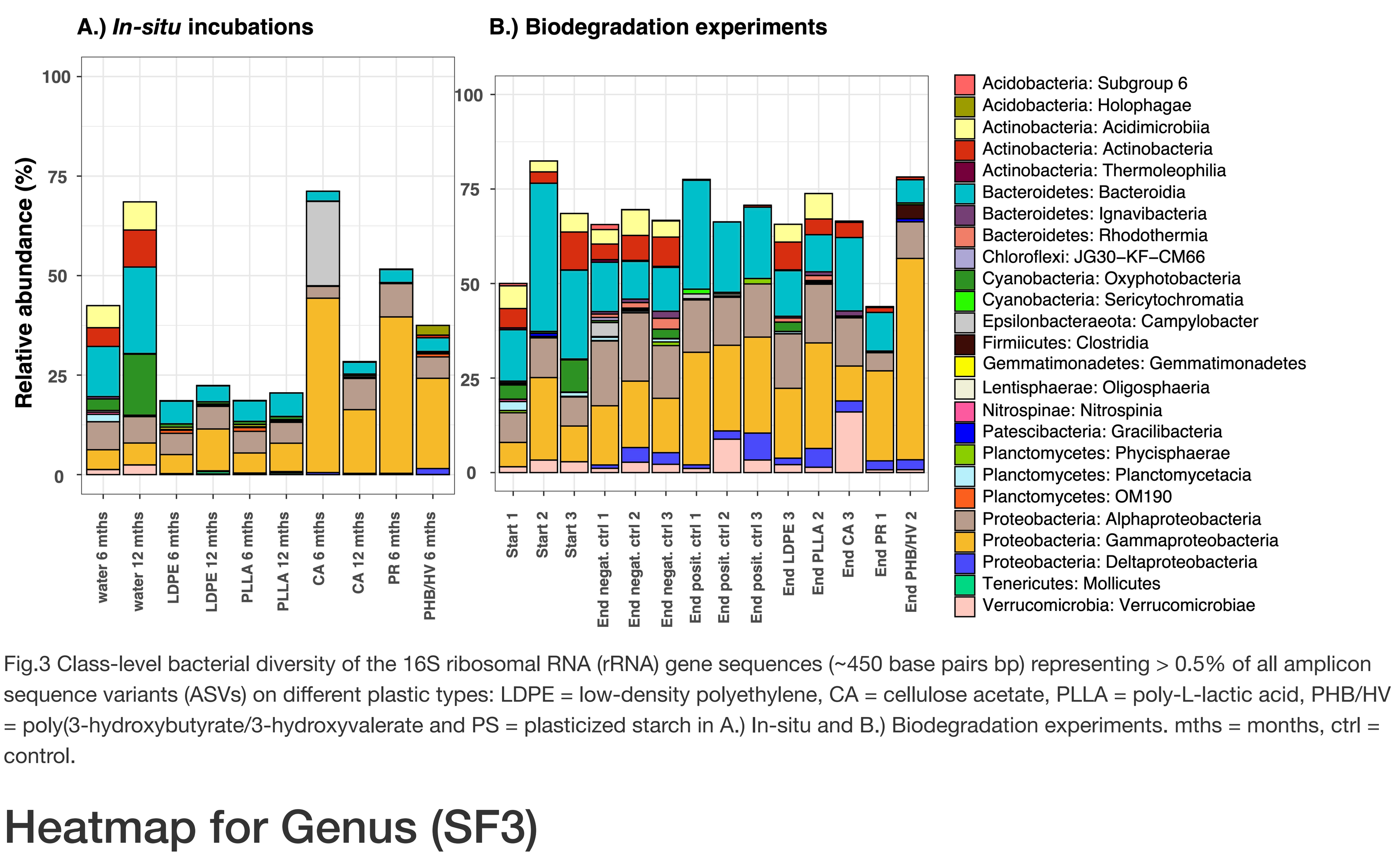

Fig.3 Class-level bacterial diversity of the 16S ribosomal RNA (rRNA) gene sequences (~450 base pairs bp) representing > 0.5% of all amplicon sequence variants (ASVs) on different plastic types: LDPE = low-density polyethylene, CA = cellulose acetate, PLLA = poly-L-lactic acid, PHB/HV = poly(3-hydroxybutyrate/3-hydroxyvalerate and PS = plasticized starch in A.) In-situ and B.) Biodegradation experiments. mths = months, ctrl = control.

Heatmap for Genus (SF3)

```
p3 <- plot_heatmap(ASVs_prop_UBI_05_Genus, "PCoA", "bray", sample.order = sampleOrder, taxa.label = "Genus", titl
e = "Merikoe", low="#000033", high="#CCFF66")
p3 + theme(axis.text.y = element_text(size=5))
```

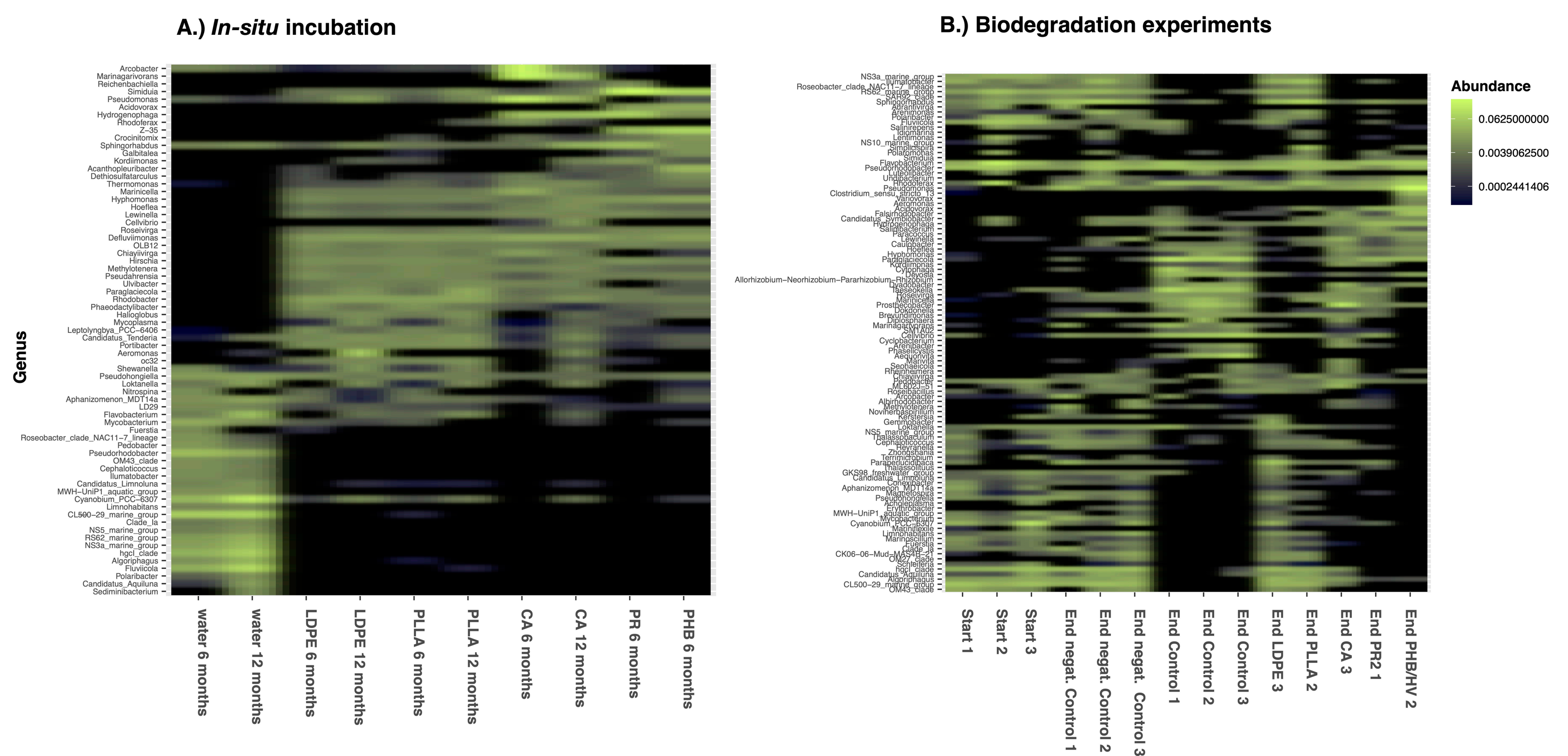

SF.3 Heatmap showing genus-level bacterial diversity of 16S ribosomal RNA (rRNA) gene sequences (~450 base pairs bp) representing > 0.5% of all amplicon sequence variants (ASVs) on different plastic types: LDPE = low-density polyethylene, CA = cellulose acetate, PLLA = poly-L-lactic acid, PHB/HV = poly(3-hydroxybutyrate/3-hydroxyvalerate and PS = plasticized starch in A.) In-situ incubations and B.) Biodegradation experiments.
